# Is There a Place for Spinal Cord Stimulation in the Management of Patients with Multiple Sclerosis? A Systematic Review of the Literature

**DOI:** 10.1155/2021/9969010

**Published:** 2021-04-19

**Authors:** Alessandro Rapisarda, Eleonora Ioannoni, Alessandro Izzo, Manuela D'Ercole, Nicola Montano

**Affiliations:** ^1^Department of Neuroscience, Neurosurgery Section, Fondazione Policlinico Universitario Agostino Gemelli IRCCS, Università Cattolica del Sacro Cuore, Rome, Italy; ^2^Neurosurgical Intensive Care Unit, Fondazione Policlinico Universitario A. Gemelli IRCCS, Rome, Italy

## Abstract

**Objective:**

Spinal cord stimulation (SCS) is a minimally invasive technique mainly used to treat neuropathic pain associated with failed back surgery syndrome. However, this therapy has been utilized to treat other chronic painful conditions, such as pain associated with multiple sclerosis (MS). Nonetheless, the efficacy of SCS in MS patients has not been fully established. In fact, in most of SCS series, MS patients represent only a subset of a bigger cohort which comprises different causes of pain, motor disorder, and other functional limitations. The aim of our study was to systematically review the literature to evaluate the effectiveness of SCS in MS patients.

**Methods:**

A literature search was performed through different databases (PubMed, Scopus, and Embase) using the following terms: “multiple sclerosis,” “spinal cord stimulation,” and “dorsal column stimulation,” according to PRISMA (Preferred Reporting Items for Systematic Reviews and Meta-analyses) guidelines.

**Results:**

A total of 452 articles were reviewed, and 7 studies were included in the present analysis. 373 MS patients were submitted to a stimulation trial, and 82 MS patients underwent a de novo implantation. 285/373 (76.4%) of cases submitted to the SCS trial were enrolled for permanent stimulation. We found a long-lasting improvement in 193/346 (55.8%) MS patients with motor disorders, in 90/134 (67.13%) MS patients with urinary dysfunction, and in 28/34 (82.35%) MS patients with neuropathic pain. The efficacy of SCS was higher for urinary dysfunction (*p* = 0.0144) and neuropathic pain (*p* = 0.0030) compared with motor disorders.

**Conclusions:**

Our systematic review evidences that SCS is effective in MS patients. Urinary dysfunction and pain symptoms seem to be most responsive to SCS. Further studies are needed to improve the patient selection and clarify the best timing to perform SCS in these patients.

## 1. Introduction

Motor disorders, neuropathic pain, and urinary dysfunction are the main causes of functional limitations in patients affected by multiple sclerosis (MS) [1–4]. All these symptoms may become unresponsive to traditional immune-modulating treatment or to medication for pain and may impact negatively on the quality of life of these patients [5]. Since the pioneering paper of Cook and Weinsten [6] who firstly submitted an MS patient to spinal cord stimulation (SCS) to treat an incoercible back pain observing a long-lasting pain relief, SCS has been used to treat the different MS-associated symptoms. Unfortunately, in most of the published papers, MS patients represent only a subset of a bigger cohort which comprises different causes of pain, motor disorder, and other functional limitations. Since few studies specifically focused on MS patients, the results of SCS in this subgroup are often ambiguous and contradictory. The aim of our study was to systematically review the literature on SCS in MS patients analysing the results of this technique on motor, pain, and urinary symptoms recovery.

## 2. Materials and Methods

### 2.1. Inclusion Criteria and Measurement of Outcomes

This study was conducted in agreement with the PRISMA (Preferred Reporting Items for Systematic Reviews and Meta-analyses) guidelines statement [7]. Three medical databases (PubMed, Scopus, and Embase) were screened for eligible scientific reports. The key words “multiple sclerosis,” “spinal cord stimulation,” and “dorsal column stimulation” were used in any possible combination. The last search was launched in June 2020. Two reviewers (A.R. and E.I.) independently screened the abstracts and the references list. Any difference was solved by consensus with a third senior author (N.M.). Studies were included if they met the following criteria: (1) English prospective or retrospective studies on SCS in MS patients, (2) series with more than 5 patients, and (3) series which clinical data, outcome, and follow-up (FU) were clearly reported for each patient. As outcome variables, motor function, pain, and urinary dysfunction were evaluated. We considered a patient improved for a specific function if the authors reported an improvement for that function regardless of the evaluation scale used.

### 2.2. Statistical Analysis

Statistical analyses were done using StatView version 5 software (SAS Institute Inc.). Statistical comparison of categorical variables was performed by *χ*2 statistic, using the Fisher's exact test. Differences were considered significant at *p* < 0.05.

## 3. Results

**Figure 1:**
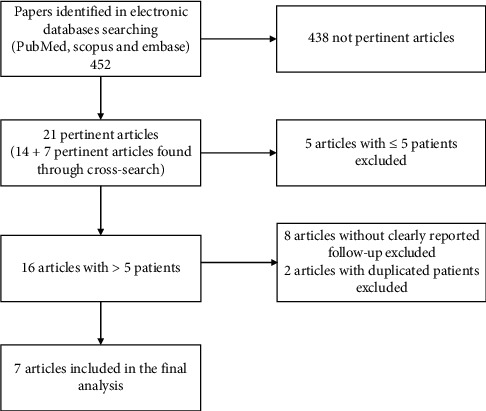
Flowchart of study selection.

A total of 452 articles were identified and reviewed (Figure 1). Finally, 7 studies were included in the present investigation (Table 1) [8–14]. Overall, there were 373 MS patients who were submitted to a SCS trial and 82 MS patients who underwent a de novo implantation. The mean age at implantation was 44.25 ± 0.75 years, and the mean FU was 44.40 ± 25.00 months. Out of the patients submitted to the SCS trial, 285/373 (76.4%) were enrolled for permanent stimulation. Overall, a long-lasting improvement (at latest available FU) was observed in 193 out of 346 (55.8%) MS patients with motor disorders, in 90 out of 134 (67.13%) MS patients with urinary dysfunction, and in 28 out of 34 (82.35%) MS patients with neuropathic pain. The efficacy of SCS was higher for urinary dysfunction (*p* = 0.0144) and neuropathic pain (*p* = 0.0030) compared with motor disorders (Table 2). These significant differences were confirmed in the subgroup of patients submitted to the SCS trial and then to the definitive implant (Table 2). In the subgroup of patients who underwent a de novo implantation (without the trial), a significant difference was maintained only for neuropathic pain (Table 2).

## 4. Discussion

MS is a chronic demyelinating disease determining a wide variety of neurological symptoms. MS has been reported as the most common cause of neurological disability in young adults [15], and its onset typically occurs between 20 and 40 years. The incidence in the female sex has been reported from two to three times higher than the male sex. The current incidence in Western Europe ranges between 2/100000/years and 18.2/100000/years [16] and is constantly increasing. MS has been categorized into four distinct clinical subtypes: relapsing-remitting, secondary-progressive, primary progressive, and progressive relapsing [17]. However, all types of MS show a neuroaxonal dysfunction determining, among the others, weakness, visual impairment, bladder dysfunction, sensory impairment, fatigue, spasticity of the extremities, trigeminal neuralgia, and neuropathic pain, which is found in approximately 50% of MS patients [18–20]. These symptoms may become unresponsive to the medical management and may significantly worsen the quality of life of these patients [2, 5, 21]. SCS is a minimally invasive technique mainly used to treat the neuropathic pain associated with the failed back surgery syndrome and other neuropathic painful conditions. Effectiveness of SCS in MS patients has been thoroughly debated in literature without a definitive conclusion. No clear indication exists on which patients can benefit from SCS and on the best timing to perform this procedure in the history of these patients. This is probably because MS patients were included in SCS series only as a subgroup of a bigger cohort of patients. The aim of our systematic review was to analyse the results of SCS in MS patients on motor function, urinary dysfunction, and neuropathic pain taking into account only articles reporting MS patients in which the results were clearly presented. We found that SCS may improve all these symptoms in more than 50% of cases and that SCS showed a significantly higher efficacy for urinary symptoms and neuropathic pain compared with motor disorders. SCS has the great advantage of being a neuromodulation procedure that is nondestructive and reversible. The main shortcoming in MS patients was the noncompatibility of the previous SCS devices with magnetic resonance imaging (MRI) which prevented the regular follow-up of the disease. However, in the last few years, the improvement of technology overcame this problem because of the development of MRI-compatible systems [21]. Moreover new stimulation paradigms, such as the high frequency paradigm, could play a role in the management of MS patients [22]. Thus, in our opinion, SCS may represent a valid option for MS patients whose symptoms are not controlled by medications. Moreover, a better selection of cases (patients who mainly complain of neuropathic pain and urinary dysfunction) and the implementation of a stimulation trial before the definitive implantation may help in increasing the number of patients responding to this treatment, as we demonstrated in this systematic review. The most frequent SCS complications comprise system malfunctioning or breakage, wound or system infections, and epidural hematoma. However, from an initial rate of 65% of the implanted patients [9], these events became less frequent subsequently [14] probably due to the evolution of the implant technique.

### 4.1. Limitations

Our study has some limitations due to the retrospective nature of data and the different evaluation scales used among the different articles. This did not allow further subgroup analyses (different MS types, different motor and urinary symptoms, and different pain locations).

## 5. Conclusions

The results of this systematic review suggest that SCS is effective in MS patients. Neuropathic pain and urinary dysfunction are significantly improved after SCS compared with motor disorders. Moreover, a proper stimulation trial is useful in increasing the number of patients responding to this treatment. Further studies with longer FU are needed to improve the patient selection, clarify the best timing to perform SCS in these patients, and better understand the potential loss of effectiveness of SCS over time.

## Figures and Tables

**Table 1:** Studies included in the pooled statistical analysis.

Author/year	Patients submitted to stimulation trial	Patients with a definitive implant	Type of the study	Follow-up (months)
Rosen and Barsoum (1979) [8]	9	9	Retrospective	6–37
Young and Goodman (1979) [9]	23	20	Retrospective	32
Illis et al. (1980) [10]	19	10	Retrospective	24
Sigfried et al. (1981) [11]	111	37	Retrospective	12–70
Cook et al. (1981) [12]	192	204 (192 posttrial + 12 de novo)	Retrospective	12
Waltz et al. (1987) [13]	None	91 (de novo)	Retrospective	6–120
Kumar et al. (2006) [14]	19	17	Retrospective	97.6

**Table 2:** Number of MS patients improved after SCS according to the evaluated function.

Function	Improvement (yes/no)	*p*
*All patients*		
Motor disorders	193/153	
Urinary dysfunction	90/44	*p* = 0.0144
Neuropathic pain	28/6	*p* = 0.0030

*Patients submitted to the trial and then implanted*		
Motor disorders	158/121	
Urinary dysfunction	40/17	*p* = 0.038
Neuropathic pain	15/4	*p* = 0.044

*Patients with de novo implantation*		
Motor disorders	35/32	
Urinary dysfunction	50/27	n.s
Neuropathic pain	13/2	*p* = 0.0126

## Data Availability

The data used to support the findings of the study are available from the corresponding author upon request.
